# Isoniazid-Resistant Tuberculosis, Taiwan, 2000–2010

**DOI:** 10.3201/eid1709.110447

**Published:** 2011-09

**Authors:** Chih-Cheng Lai, Che-Kim Tan, Yu-Tsung Huang, Chun-Hsing Liao, Po-Ren Hsueh

**Affiliations:** Author affiliations: Chi-Mei Medical Center, Liouying, Tainan, Taiwan (C.C. Lai), Chi-Mei Medical Center, Yong-Kang, Tainan (C.-K. Tan);; National Taiwan University Hospital, Taipei, Taiwan (Y.-T. Huang, P.-R. Hsueh);; National Taiwan College of Medicine, Taipei (Y.-T. Huang, P.-R. Hsueh);; Far Eastern Memorial Hospital, New Taipei City, Taiwan (C.-H. Liao)

**Keywords:** isoniazid-resistant, drug resistance, tuberculosis and other mycobacteria, Taiwan, letter

**To the Editor:** Vinnard et al. (*1*) reported that the risk factors associated with initial isoniazid resistance among patients with tuberculous meningitis in the United States during 1993–2005 included young age (25–34 years) and foreign birth (*1*). In a previous survey, conducted in Taiwan during 2000–2008, we found the rate of antituberculosis drug resistance to be lower for older patients than for younger patients (*2*); however, current information about the patient characteristics associated with isoniazid-resistant tuberculosis (TB) in Taiwan is lacking. Therefore, to determine the risk factors associated with initial isoniazid resistance among patients with TB in Taiwan, we conducted a retrospective study.

The study was conducted at the National Taiwan University Hospital, a 2,500-bed tertiary care center in northern Taiwan. We analyzed culture-confirmed *Mycobacterium tuberculosis* isolates obtained from hospitalized patients during January 2000–December 2010. A nonduplicate isolate was defined as 1 isolate collected for evaluation from 1 patient who visited the hospital (as inpatient or outpatient). If multiple isolates were available from a patient, only the one first isolated was analyzed. All specimens were processed and pretreated as described elsewhere (*3*). Patients with multidrug-resistant TB were excluded on the basis of evidence for differences in the epidemiology of isoniazid-resistant (rifampin-susceptible) TB and multidrug-resistant TB (*4*). Immigrant populations in Taiwan are limited; therefore, we did not analyze the origin of the patients.

After excluding patients with multidrug-resistant TB, we analyzed 4,289 nonduplicate isolates, of which 3,842 (89.6%) were susceptible to isoniazid and the other 447 (10.4%) were resistant to isoniazid. In terms of demographic associations, patients 34–<44 years of age were more likely than those >74 years of age to have an isoniazid-resistant strain (Table). In addition, patients with extrapulmonary TB were less likely than patients with pulmonary TB to be infected with isoniazid-resistant TB. We also identified 34 patients with TB meningitis. After excluding 2 patients with multidrug-resistant TB, we found that 31 patients (mean age 56.6 years) had isoniazid-susceptible TB meningitis and a 50-year-old man had meningitis caused by isoniazid-resistant TB.

**Table:** Factors associated with isoniazid resistance among *Mycobacterium tuberculosis* isolates, National Taiwan University Hospital, Taiwan, 2000–2010*

Factor	No. infections	No. isoniazid- resistant infections	Resistance rate	OR (95% CI)
Patient age, y				
<14	42	2	4.76	0.32 (0.08–1.23)
14 to <24	241	22	9.13	0.64 (0.42–1.07)
24 to <34	342	36	10.53	0.75 (0.48–1.18)
34 to <44	384	52	13.54	Reference
44 to <54	490	56	11.43	0.82 (0.55–1.23)
54 to <64	609	70	11.49	0.83 (0.57–1.22)
64 to <74	845	96	11.36	0.82 (0.57–1.17)
74 to <84	986	85	8.62	0.60 (0.42–0.87)
84	350	28	8.00	0.56 (0.34–0.90)
Patient sex				
F	1,356	128	9.44	0.85 (0.69–1.06)
M	2,933	319	10.88	Reference
Pulmonary tuberculosis				
No	772	56	7.25	0.63 (0.47–0.84)
Yes	3,517	391	11.12	Reference

*OR, odds ratio; CI, confidence interval.

Our results are in agreement with those reported in a previous study in the United States, which found that the rate of isoniazid resistance was lower for isolates from elderly patients (*1**,**4*). This phenomenon may be attributable to the reactivation of a dormant infection. Because isoniazid was introduced to Taiwan for the treatment of TB in 1952, elderly persons in Taiwan probably did not receive isoniazid if their TB developed when they were young. In the present study, the resistant rate was lower for *M. tuberculosis* strains isolated from elderly persons than from younger adults. These findings suggest that first-line anti-TB medications still have good in vitro activity against *M. tuberculosis* strains in elderly patients.

In contrast to the study by Vinnard et al. (*1*), our results showed that isoniazid-resistant *M. tuberculosis* was significantly less likely to be isolated from nonrespiratory than from respiratory specimens. The reasons for this finding are unclear. Continuous monitoring of antimicrobial drug resistance among *M. tuberculosis* isolates isolated from various body sites needs to be incorporated into any TB surveillance program.

Gathering data on drug resistance rates is a major aspect of the global TB control program. Clinicians must have knowledge of local epidemiology, and mycobacteriology laboratories should maintain up-to-date information on drug susceptibility test profiles of local *M. tuberculosis* isolates.
